# The association between metabolic syndrome and the risk of prostate cancer, high-grade prostate cancer, advanced prostate cancer, prostate cancer-specific mortality and biochemical recurrence

**DOI:** 10.1186/1756-9966-32-9

**Published:** 2013-02-13

**Authors:** Yu-zhu Xiang, Hui Xiong, Zi-lian Cui, Shao-bo Jiang, Qing-hua Xia, Yong Zhao, Guan-bin Li, Xun-bo Jin

**Affiliations:** 1 Minimally Invasive Urology Center, Provincial Hospital Affiliated to Shandong University, Jinan, 250021, China

**Keywords:** Gleason score, Recurrence, Clinical stage, Aggressiveness

## Abstract

**Background:**

Although a previous meta-analysis reported no association between metabolic syndrome (MetS) and prostate cancer risk, a number of studies suggest that MetS may be associated with the aggressiveness and progression of prostate cancer. However, these results have been inconsistent. This systematic review and meta-analysis investigated the nature of this association.

**Methods:**

We systematically searched MEDLINE, EMBASE and bibliographies of retrieved studies up to January 2013 using the keywords “metabolic syndrome” and “prostate cancer”. We assessed relative risks (RRs) of the prostate cancer, several parameters of prostate cancer aggressiveness and progression associated with MetS using 95% confidence intervals (95% CIs).

**Results:**

The literature search produced 547 hits from which 19 papers were extracted for the meta-analysis. In cancer-free population with and without MetS, the combined adjusted RR (95% CI) of prostate cancer risk and prostate cancer-specific mortality in longitudinal cohort studies is 0.96 (0.85 ~ 1.09) and 1.12 (1.02 ~ 1.23) respectively. In the prostate cancer patients with and without MetS, the combined unadjusted OR (95% CI) of high grade Gleason prostate cancer is 1.44 (1.20 ~ 1.72), the OR of advanced prostate cancer is 1.37 (1.12 ~ 1.68) and the OR of biochemical recurrence is 2.06 (1.43 ~ 2.96).

**Conclusions:**

The overall analyses revealed no association between MetS and prostate cancer risk, although men with MetS appear more likely to have high-grade prostate cancer and more advanced disease, were at greater risk of progression after radical prostatectomy and were more likely to suffer prostate cancer-specific death. Further primary studies with adjustment for appropriate confounders and larger, prospective, multicenter investigations are required.

## Background

In men, prostate cancer (PCa) is the most frequently diagnosed malignancy in industrialized countries [1] and it is the second most commonly diagnosed cancer and the sixth leading cause of cancer death worldwide [2]. There is a clear need for a better understanding of the risk factors related to PCa development and progression. Age, race and family history are the only established prostate cancer risk factors and these factors are all non-modifiable. Recently, modifiable lifestyle factors such as physical activity and diet have been investigated. Because a higher incidence of PCa was associated with a higher prevalence of “western” lifestyle, it has been suggested that these lifestyle factors play a significant role in the pathogenesis of PCa [3].

Metabolic syndrome (MetS) is a cluster of cardiovascular risk factors that includes hypertension, diabetes mellitus, obesity, hypertriglyceridemia, and low high-density lipoprotein cholesterol, with insulin resistance as the underlying hallmark feature [4]. The prevalence of MetS has been increasing worldwide and has become a major public health problem in many western countries. For example, 35%-41% of adults in the USA are reported to exhibit MetS [5]. Recently, increasing evidences suggests that MetS may be involved in the development and progression of certain types of cancer as an independent etiologic factor including breast cancer [6], endometrial cancer [7], colorectal cancer [8], pancreatic cancer [9] and prostate cancer [10]. MetS was firstly observed as a composite factor associated with prostate cancer risk in 2004 [11], and more studies have since reported the association between MetS and prostate cancer. However, the studies investigating the association between MetS and prostate cancer risk have reported inconsistent findings [12-21].

It is crucial to review and evaluate the magnitude to which MetS affects the development and progression of PCa, as proper management of this modifiable lifestyle factor may help improve PCa outcomes.

A recently performed meta-analysis study summarized the association between MetS and the incidence of some common cancer types, including prostate cancer. The results, based on 14 databases, revealed that MetS was not associated with prostate cancer risk [22]. However, a new investigation on MetS and prostate cancer risk was published recently [19], and much increasing evidence in the latest investigations suggests that MetS may be associated with the aggressiveness and progression of PCa; prostate cancer patients with MetS may suffer more aggressive disease and adverse clinical outcomes [19,23-27]. However, inverse results [28] or no significant associations [14,20,29,30] have been reported in other studies. Therefore, to thoroughly investigate the nature of this association, we focused on longitudinal cohort studies and conducted a new meta-analysis to confirm the association between MetS and prostate cancer risk by searching the latest literature. Subsequently, we performed another meta-analysis to quantitatively summarize several parameters of PCa aggressiveness and progression, including Gleason score, clinical stage, biochemical recurrence and prostate cancer-specific mortality associated with MetS.

## Methods

### Search strategy

We systematically searched MEDLINE, EMBASE through January 2013 for human studies on the association between MetS and PCa with the following medical subject heading terms and/or text words: “metabolic syndrome”, “insulin resistance syndrome”, or “syndrome X”, combined with “prostate cancer”, “prostatic cancer”, “prostate neoplasm”, or “prostatic neoplasm”. We also manually searched relevant journals, bibliographies, and reviews for additional articles. The search had no language restriction.

### Inclusion criteria

The eligibility of each study was assessed independently by two investigators (YX and HX). We included only cohort studies of MetS and prostate cancer risk or prostate cancer-specific mortality and clinical studies of MetS and Gleason score or clinical stage at diagnosis or biochemical recurrence after treatment. We included studies that reported standardized forms of relative risk, risk ratio, hazard ratio or odds ratio with estimates of confidence intervals (CIs) or with sufficient data to estimate CIs. We used relative risks (RRs) to represent various effect estimates in a cohort study in this meta-analysis.

### Exclusion criteria

We excluded reviews, editorials, meta-analysis and animal studies. Among the 23 studies that underwent full-text reviews, we excluded a study on MetS and prostate cancer risk of re-biopsy [31], a study that did not use a standard definition of MetS [32,33] and one case-control study on MetS and prostate cancer risk [21]. For studies previously published on the same database [34,35], we included only the most recent findings [19,20]. All of the studies on which we focused reported RRs with 95% CIs or sufficient data to estimate them.

### Data extraction

The data extracted included publication data (the first author’s last name, year of publication, and country of the population studied), study design, population resources, number of cases, risk estimates with their corresponding CIs, and variables controlled for by matching or in the most adjusted model. Abstractions of the data elements were conducted separately by two authors; discordant results were resolved by consensus.

### Statistical analysis

Firstly, we updated the data and attempted to analyze the association of MetS with the prostate cancer risk in longitudinal cohort studies only. Subsequently, we assessed the association between MetS and prostate cancer-specific mortaligy in cohort studies and between MetS and high grade Gleason PCa and/or advanced PCa or biochemical recurrence in clinical studies. We pooled all of the RRs for MetS and assessed the heterogeneity between the studies by Q and I^2^ statistics, which are distributed as x^2^ statistics [36]. A value of P < 0.10 was used to indicate lack of homogeneity (heterogeneity) among effects. We used a fixed-effects model if I^2^ value significance was <0.1; otherwise, we used a random-effect model. Sensitivity analysis was conducted by omitting one study at a time, generating the pooled estimates and comparing with the original estimates. Funnel plots and both Begg’s and Egger’s tests were used to evaluate publication bias. All analyses were performed using STATA version 9.0 statistical software (Stata, College Station, Texas, USA). All statistical comparisons were 2-sided, and a p-value < 0.05 was considered statistically significant).

## Results

### Study characteristics

Nineteen studies met the search inclusion and exclusion criteria. The characteristics of included studies are presented in Tables 1 and 2.

**Table 1:** Characteristics of cohort studies of metabolic syndrome and prostate cancer risk

**Author yr (ref. no.)**	**Country**	**Population**	**Mean age, yr**	**Mean FU time, yr**	**Time period**	**Cohort size**	**Definition of MetS**	**No. of cases**	**RRs**	**95% CI**	**Controlled variables**
Laukkanen 2004 [11]	Finland	Kuopio communities	52.6	15	1984-2001	1,880	WHO	56	RR 1.90	1.1-3.5	Age
Tande 2006 [12]	United States	ARIC* (49% white, 51% African American)	45-64	12.1	1987-2000	6,429	NCEP-ATP-III	385	RR 0.77	0.60-0.98	Age, race
Russo 2008 [13]	Italy	A pharmacologically based diagnosis	40	2.7	1999-2005	NA	A pharmacologically based diagnosis	94	RR 0.93	0.75-1.14	Age
Martin 2009 [14]	Norway	HUNT2	48 ± 16.4	9.3	1996-2005	29,364	NCEP-ATP-III	687	RR 0.91	0.77-1.09	Age+
Inoue 2009 [15]	Japan	Japan PHC population	40-69	10.2	1993-2004	9,548	IDF	119	HR 0.76	0.47-1.22	Age+
Grundmark 2010 [16]	Sweden	ULSAM	50	30.3	1970-2003	2,183	NCEP-ATP-III	226	RR 1.29	0.89-1.88	Age
						2,287	IDF	234	RR 1.18	0.81-1.71	
Wallner 2010 [17]	United States	Olmsted County	40-79	15	1990-NA	2,445	WHO	206	HR 0.65	0.37-1.10	Age
Osaki 2011 [18]	Japan	The population-based cancer registry	60.5 ± 10.8	9.3	1992-2007	8,239	NCEP-ATP-III	152	HR 1.37	0.91-2.06	Age
						8,239	IDF	152	HR 1.18	0.74-1.90	
Häggström 2012 [19]	Norway	Me-Can	44	12	NA	289,866	Upper quartile levels ATP-III criteria	6,922	RR 0.96	0.92-1.00	Age+
	Sweden										
	Austria										

*MetS* = metabolic syndrome; *PCa* = prostate cancer; *RRs* = Relative risks; *CI* = confidence interval; *Age* + =At least age; *WHO* = World Health Organization; *NCEP-ATP-III* = National Cholesterol Education Program Adult Treatment Panel III; *IDF* = International Diabetes Federation; *HUNT 2* = Nord-Trondelang Health Study; *ARIC* = Atherosclerosis Risk in Communities; *OR* = odds ratio; *We use White-American data.

**Table 2:** Characteristics of studies of metabolic syndrome and parameters of prostate cancer

**Author yr (ref. no.)**	**Country**	**Study design**	**Population**	**Mean age,yr**	**Time period**	**Definition Vof MetS**	**No. of cases**	**Outcomes**	**RRs**	**95% CI**
B.K 2007 [29]	Korea	Cross-section study	Patients who underwent radical retropubic prostatectomy	64.8 ± 6.2	2004-2006	NCEP-ATP-III	261	Gleason score ≥7(4 + 3)	0.972	0.637-1.482
								Clinical stage ≥ T3	0.991	0.532-1.846
Beebe-Dimmer 2009 [20]	United States	Case-control study	GECAP	62.3	1999-2004	NCEP-ATP-III	637	Gleason score ≥7(4 + 3)	1.2	0.64-2.27
								Clinical stage ≥ T3	1.17	0.55-2.51
Castillejos-Molina 2011 [23]	Mexico	Case-control study	Patients with PC who underwent surgical treatment	64.8 ± 6.97	1990-2007	WHO	210	Gleason score >7	3.346	1.144-9.791
								Clinical stage ≥ T3	1.628	0.915-2.896
Kheterpal 2012 [24]	United States	Cross-section study	Patients who underwent robot assisted radical prostatectomy	60.7 ± 6.9	2005-2008	IDF	2756	Gleason score ≥7(4 + 3)	1.328	0.978-1.802
								Clinical stage ≥ T3	1.416	1.109-1.808
De Nunzio 2011 [25]	Italy	Cross-section study	Patients who underwent prostate biopsy for PSA > 4 ng/ml or abnormal DRE	69	2009-2011	NCEP-ATP-III	83	Gleason score ≥7	3.82	1.33-10.9
								Clinical stage ≥ T3	NA	NA
Jeon 2012 [28]	Korea	Cross-section study	Patients who underwent prostate biopsy for PSA > 4 ng/ml or abnormal DRE	68.86 ± 8.95	2003-2011	NCEP-ATP-III	90	Gleason score ≥7(4 + 3)	0.101	0.022-0.473
								Clinical stage ≥ T3	NA	NA
Morote 2012 [26]	Spain	Cross-section study	Patients who underwent prostate biopsy for PSA > 4 ng/ml or abnormal DRE	68(46-79)	2006-2010	NCEP-ATP-III	848	Gleason score >7	1.75	1.260-2.414
								Clinical stage ≥ T3	NA	NA
Castillejos-Molina 2011 [23]	Mexico	Case-control study	Patients with PC who underwent surgical treatment	64.8 ± 6.97	1990-2007	WHO	210	Biochemical recurrence	2.73	1.65-4.50
Post 2011 [27]	United States	Case-control study	Patients who underwent radical prostatectomy	60.9	1999- 2004	NCEP-ATP-III	383	Biochemical recurrence	1.5	0.90-2.6
Jaggers 2009 [30]	United States	Cohort study	Aerobics Center Longitudinal Study	20-88	1977-2003	NCEP-ATP-III	185	Mortality	1.32	0.63-2.77
Martin 2009 [14]	Norway	Cohort study	HUNT2	48 ± 16.4	1996-2005	NCEP-ATP-III	107	Mortality	0.81	0.52-1.25
Häggström 2012 [19]	Norway Sweden Austria	Cohort study	Me-Can	44	NA	Upper quartile Levels ATP-III criteria	961	Mortality	1.13	1.03-1.25

*PCa* = prostate cancer; *RRs* = Relative risks; *CI* = confidence interval; *WHO* = World Health Organization; *NCEP-ATP-III* = National Cholesterol Education Program Adult Treatment Panel III; *IDF* = International Diabetes Federation; *HUNT 2* = Nord-Trondelang Health Study; *NA* = Not available; *DRE* = Digital rectal examination.

**Figure 1:**
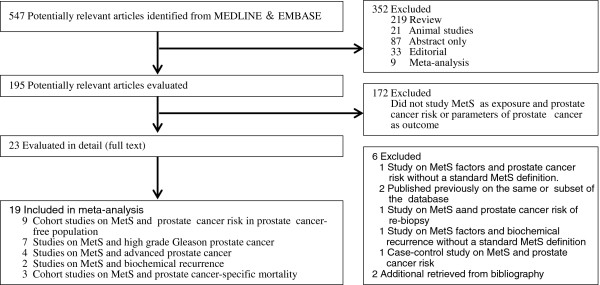
Selection of studies for meta-analysis.

Detailed search steps are described in Figure 1. Briefly, from the initial literature search we identified 547 abstracts. Twenty-three articles were considered of interest and full text of each article was retrieved for detailed evaluation. Eleven studies investigated the association between MetS and prostate cancer [11-21]. Nine of them were longitudinal cohort studies that reported the RRs of PCa in cancer-free population with and without MetS [7-15]. Seven studies evaluated MetS and pathological and clinical stages of PCa, of these studies, 7/7 investigate Gleason score [20,23-26,28,29] and 4/7 investigated clinical stage [20,23,24,29]. Two case-control studies explored biochemical recurrence after primary treatment [23,27], and three longitudinal cohort studies focused on prostate cancer-specific mortality [14,19,30].

### Main findings

#### Prostate cancer risk

**Figure 2:**
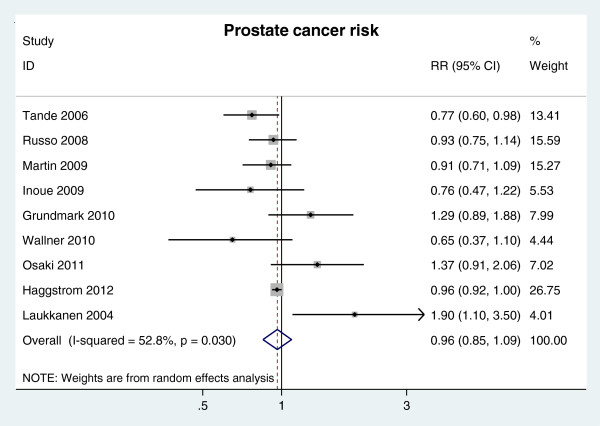
RR of prostate cancer risk for MetS presence.

Result from a meta-analysis based on nine longitudinal cohort studies revealed that there was no association between MetS and prostate cancer risk (RR = 0.96, 95% CI 0.85-1.09 n = 9 studies) (Figure 2).

#### Prostate cancer aggressiveness

##### High grade Gleason score

**Figure 3:**
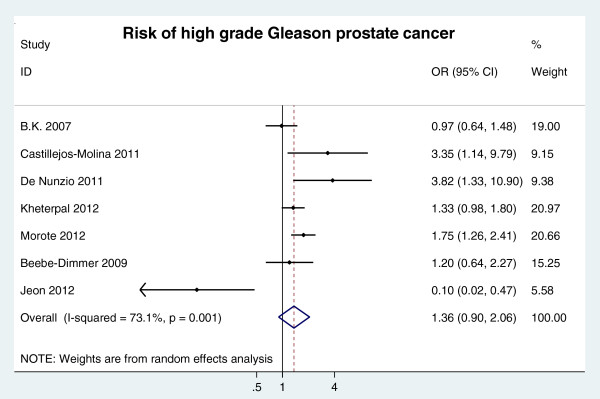
RR of high grade Gleason prostate cancer risk for MetS presence.

The definition of high grade Gleason score is ≥ 7 or > 7. A trend for a 36% increased risk of a high Gleason score in patients with MetS (OR = 1.36, 95% CI 0.90-2.06 n = 7 studies) was identified based on a meta-analysis of seven total relative databases (Figure 3).

##### Advanced clinical stage

**Figure 4:**
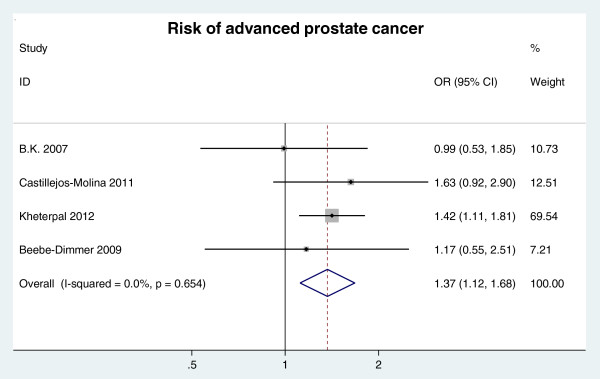
RR of advanced clinical stage for MetS presence.

Advanced clinical stage was defined as a clinical stage ≥ T3. Four databases were included in the analysis of the association of MetS with advanced clinical stage. The analysis revealed that MetS was significantly associated with a 37% increased risk of advanced clinical stage (OR = 1.37, 95% CI: 1.12 ~ 1.68; n = 4 studies) (Figure 4).

#### Prostate cancer progression

##### Biochemical recurrence

**Figure 5:**
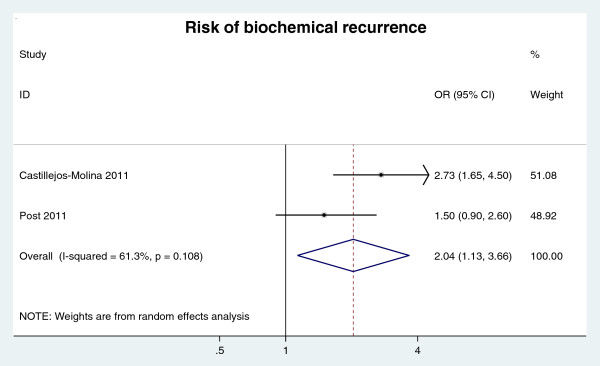
RR of biochemical recurrence for MetS presence.

Only two databases [23,27] focused on the association of MetS which biochemical recurrence. The Individual study results and the overall summary results are presented in Figure 5. The result indicates that MetS was significantly associated with 2-folds of increased risk of biochemical recurrence (OR = 2.06, 95% CI: 1.43-2.96, n = 2 studies).

##### Prostate cancer-specific mortality

**Figure 6:**
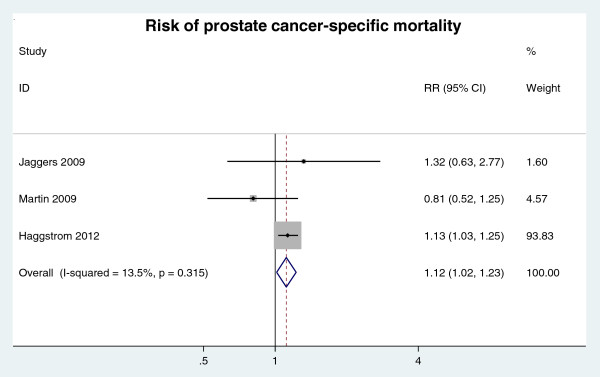
RR of prostate cancer-specific mortality for MetS presence.

Three cohort studies [14,19,30] investigated how MetS affected prostate cancer-specific mortality. The meta-analysis revealed that MetS was significantly associated with a higher risk of the prostate cancer-specific death (RR = 1.12, 95% CI: 1.02 ~ 1.23; n = 3 studies) (Figure 6).

### Sensitivity analysis

We conducted sensitivity analysis by omitting one study at a time, generating the pooled estimates and comparing the pooled estimates with the original estimates. Omitting any one of nine studies concerning MetS and prostate cancer risk or omitting any one of four studies concerning MetS and advanced clinical stage produced no dramatic influence on the original pooled RRs. Omitting Jeon 2012 database [28] in the 7 studies concerning MetS and Gleason score produced a significant OR = 1.44 (95% CI: 1.20 ~ 1.72), whereas none of the remaining severn studies exhibited a significant influence on the original estimates. For biochemical recurrence and prostate cancer-specific mortality, there were too few studies to do a sensitivity analysis.

### Publication bias

**Figure 7:**
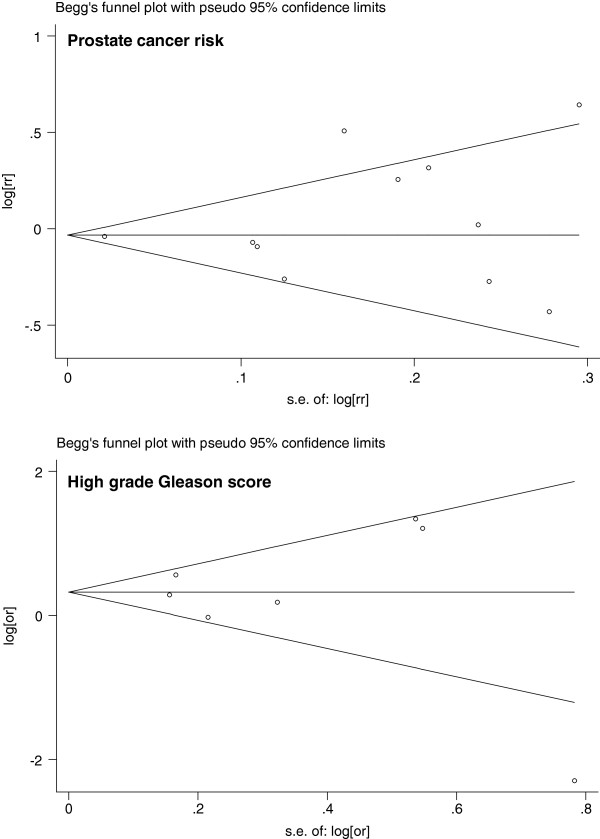
Funnel plot with pseudo 95% confidence limits.

Visual inspection of the Begg funnel plot for both PCR and Gleason score did not reveal the asymmetry typically associated with publication bias (Figure 7). Evidence of publication bias was also not seen with the Egger or Begg tests (Egger P = 0.27 and 0.64 for prostate cancer risk and Gleason score respectively).

## Discussion

In 2007, Hsing et al. summarized five studies on MetS and prostate cancer risk and concluded that the epidemiologic evidence was insufficient to suggest a link between MetS and PCa [37]. In 2012, Esposito et al. performed a systematical review and meta-analysis on the association of MetS and cancer risk including prostate cancer. The authors also concluded that MetS was not associated with prostate cancer risk too [22]. In the present study, we updated the data and used the current evidence to analyze whether MetS is associated with prostate cancer risk. We observed the same result as previous meta-analysis; no association could be detected between Mets and prostate cancer.

We believe the result is reliable for two reasons. Firstly, only longitudinal cohort studies were included in this analysis, imparting strong evidence for our conclusions. In addition, the association between MetS and prostate cancer may be affected by several factors, including heterogeneity among the individual studies. The heterogeneity may arise from differences in age, race, the definition of MetS [22], and geographic factors [26]. Further, MetS is a syndrome composed of at least 3 components, and the individual component may exert antagonistic functions on one another Thus the syndrome may represent an integrated outcome that combines neutralizing positive and negative functions. For example, a meta-analysis revealed that diabetes mellitus was significantly negatively associated with prostate cancer risk in population-based studies (RR = 0.72, 95% CI: 0.64-0.81) and cohort studies conducted in the USA (RR = 0.79, 95% CI: 0.73, 0.86) [38]. Furthermore, several genome-wide association studies suggest that diabetes mellitus and prostate cancer share certain genetic factors, including the *HNF1β* and *JAZF1* genes, and a previous study suggested that *JAZF1* might represent a potential target against diabetes and obesity [39]. Although hypertension was found to be positively associated with prostate cancer risk [33,40-42], Obesity is negatively with localized prostate cancer (0.94, 95% CI, 0.91-0.97) and positively associated with advanced prostate cancer risk (1.07, 95% CI 1.01-1.13) [43].

However, after analyses of several parameters of PCa aggressiveness and progression, we found MetS to be significantly associated with an increased risk of prostate cancer with a high-Gleason score or advanced clinical stage, with biochemical recurrence after primary treatment and with prostate cancer-specific mortality. If confirmed by more investigations, this finding may open a new research field on PCa development and progression, potentially leading to new strategies or methods for PCa treatment. MetS is a major public health problem and prostate cancer is the most prevalent solid organ tumor, accounts for 29% of all cancer cases and the second most common cause of death by cancer among men in the USA [44]. Therefore we believe that there is a compelling need to investigate this association between MetS and prostate cancer although the association is not strong.

Nevertheless, the reliability of these results is limited. First, Gleason score and clinical stage data were extracted from cross-sectional studies not longitudinal cohort studies. Second, there exists a small difference among studies on the definition of high-grade Gleason PCa, some authors defined a high Gleason score ≥ 7 whereas others defined a high score as >7. Third, the pathological stage data in some studies were from biopsy not radical prostatectomy specimens. Last but not least, to date there remains limited studies focusing on this association, although many of the available studies are well designed case-control or longitudinal cohort studies.

In addition to the limitations listed above, another limitation for the analyses of the association between MetS and prostate cancer risk or prostate cancer parameters is that we did not perform a meta-regression to attempt to explain the heterogeneity of the study because of the varying adjustments in the individual studies. The result of a recent meta-analysis on 9 cross-sectional studies of metabolic syndrome in adult cancer survivors increases the weight of this suspicion, as it revealed that no significant association was found for non-hematologic malignancies, including testicular tumor, prostate cancer, sarcoma, and epithelial ovarian [45]. Therefore, there is an urgent future need to confirm this association and to find potential mechanisms to explain how metabolic factors affect the development or progression of PCa.

## Conclusions

Based on the current findings, MetS is not associated with prostate cancer risk, but preliminary evidences demonstrates that men with MetS more frequently suffer high-grade prostate cancer, more advanced disease and are at greater risk of progression after radical prostatectomy and prostate cancer-specific death. Together, these findings indicate that MetS may be associated with the progression of prostate cancer and adverse clinical outcomes. Further studies with adjustment for appropriate confounders and larger, prospective, multicenter investigations are required in the future.

## Abbreviations

PCa: Prostate cancer; MetS: Metabolic syndrome; RR: Relative risk; OR: Odd ratio; HR: Hazard ratio; CIs: Confidence intervals.

## Competing interests

No potential conflicts of interest were disclosed.

## Authors’ contributions

This study was designed and supervised by XJ. Literature search, selection and data extraction was by YX and HX, and data analyses were performed by YX, HX, ZC, SJ, QX, YZ and GL. Data interpretation and manuscript writing received contributions from all authors. All authors read and approved the final manuscript.

## References

[B1] Jemal A Bray F Center MM Ferlay J Ward E Forman D Global cancer statistics CA Cancer J Clin 2011 61 2 69 90 10.3322/caac.20107 21296855

[B2] Siegel R Ward E Brawley O Jemal A Cancer statistics, 2011: the impact of eliminating socioeconomic and racial disparities on premature cancer deaths CA Cancer J Clin 2011 61 4 212 236 10.3322/caac.20121 21685461

[B3] Nelson WG De Marzo AM Isaacs WB Prostate cancer N Engl J Med 2003 349 4 366 381 10.1056/NEJMra021562 12878745

[B4] Reaven GM Banting lecture 1988. Role of insulin resistance in human disease Diabetes 1988 37 12 1595 1607 10.2337/diabetes.37.12.1595 3056758

[B5] Alberti KG Eckel RH Grundy SM Zimmet PZ Cleeman JI Donato KA Fruchart JC James WP Loria CM Smith SC Jr Harmonizing the metabolic syndrome: a joint interim statement of the International Diabetes Federation Task Force on Epidemiology and Prevention; National Heart, Lung, and Blood Institute; American Heart Association; World Heart Federation; International Atherosclerosis Society; and International Association for the Study of Obesity Circulation 2009 120 16 1640 1645 10.1161/CIRCULATIONAHA.109.192644 19805654

[B6] Pothiwala P Jain SK Yaturu S Metabolic syndrome and cancer Metab Syndr Relat Disord 2009 7 4 279 288 10.1089/met.2008.0065 19284314 PMC3191378

[B7] Rosato V Zucchetto A Bosetti C Dal Maso L Montella M Pelucchi C Negri E Franceschi S La Vecchia C Metabolic syndrome and endometrial cancer risk Ann Oncol 2011 22 4 884 889 10.1093/annonc/mdq464 20937645

[B8] Pelucchi C Negri E Talamini R Levi F Giacosa A Crispo A Bidoli E Montella M Franceschi S La Vecchia C Metabolic syndrome is associated with colorectal cancer in men Eur J Cancer 2010 46 10 1866 1872 10.1016/j.ejca.2010.03.010 20395126

[B9] Rosato V Tavani A Bosetti C Pelucchi C Talamini R Polesel J Serraino D Negri E La Vecchia C Metabolic syndrome and pancreatic cancer risk: a case-control study in Italy and meta-analysis Metabolism 2011 60 10 1372 1378 10.1016/j.metabol.2011.03.005 21550085

[B10] Zhou JR Blackburn GL Walker WA Symposium introduction: metabolic syndrome and the onset of cancer Am J Clin Nutr 2007 86 3 s817 s819 18265474 10.1093/ajcn/86.3.817S PMC4144325

[B11] Laukkanen JA Laaksonen DE Niskanen L Pukkala E Hakkarainen A Salonen JT Metabolic syndrome and the risk of prostate cancer in Finnish men: a population-based study Cancer Epidemiol Biomarkers Prev 2004 13 10 1646 1650 15466982

[B12] Tande AJ Platz EA Folsom AR The metabolic syndrome is associated with reduced risk of prostate cancer Am J Epidemiol 2006 164 11 1094 1102 10.1093/aje/kwj320 16968859

[B13] Russo A Autelitano M Bisanti L Metabolic syndrome and cancer risk Eur J Cancer 2008 44 2 293 297 10.1016/j.ejca.2007.11.005 18055193

[B14] Martin RM Vatten L Gunnell D Romundstad P Nilsen TI Components of the metabolic syndrome and risk of prostate cancer: the HUNT 2 cohort, Norway Cancer Causes Control 2009 20 7 1181 1192 10.1007/s10552-009-9319-x 19277881

[B15] Inoue M Noda M Kurahashi N Iwasaki M Sasazuki S Iso H Tsugane S Impact of metabolic factors on subsequent cancer risk: results from a large-scale population-based cohort study in Japan Eur J Cancer Prev 2009 18 3 240 247 10.1097/CEJ.0b013e3283240460 19491612

[B16] Grundmark B Garmo H Loda M Busch C Holmberg L Zethelius B The metabolic syndrome and the risk of prostate cancer under competing risks of death from other causes Cancer Epidemiol Biomarkers Prev 2010 19 8 2088 2096 10.1158/1055-9965.EPI-10-0112 20647401 PMC2923431

[B17] Wallner LP Morgenstern H McGree ME Jacobson DJ St Sauver JL Jacobsen SJ Sarma AV The effects of metabolic conditions on prostate cancer incidence over 15 years of follow-up: Results from the Olmsted County Study BJU Int 2011 107 6 929 935 10.1111/j.1464-410X.2010.09703.x 20880183 PMC3099535

[B18] Osaki Y Taniguchi S Tahara A Okamoto M Kishimoto T Metabolic syndrome and incidence of liver and breast cancers in Japan Cancer Epidemiol 2012 36 2 141 147 10.1016/j.canep.2011.03.007 21890443

[B19] Haggstrom C Stocks T Ulmert D Bjorge T Ulmer H Hallmans G Manjer J Engeland A Nagel G Almqvist M Prospective study on metabolic factors and risk of prostate cancer Cancer 2012 118 24 6199 6206 10.1002/cncr.27677 23090855

[B20] Beebe-Dimmer JL Nock NL Neslund-Dudas C Rundle A Bock CH Tang D Jankowski M Rybicki BA Racial differences in risk of prostate cancer associated with metabolic syndrome Urology 2009 74 1 185 190 10.1016/j.urology.2009.03.013 19428088 PMC2704922

[B21] Pelucchi C Serraino D Negri E Montella M Dellanoce C Talamini R La Vecchia C The metabolic syndrome and risk of prostate cancer in Italy Ann Epidemiol 2011 21 11 835 841 10.1016/j.annepidem.2011.07.007 21982487

[B22] Esposito K Chiodini P Colao A Lenzi A Giugliano D Metabolic Syndrome and Risk of Cancer: A systematic review and meta-analysis Diabetes Care 2012 35 11 2402 2411 10.2337/dc12-0336 23093685 PMC3476894

[B23] Castillejos-Molina R Rodriguez-Covarrubias F Sotomayor M Gomez-Alvarado MO Villalobos-Gollas M Gabilondo F Feria-Bernal G Impact of metabolic syndrome on biochemical recurrence of prostate cancer after radical prostatectomy Urol Int 2011 87 3 270 275 10.1159/000329280 21876327

[B24] Kheterpal E Sammon JD Diaz M Bhandari A Trinh QD Pokala N Sharma P Menon M Agarwal PK Effect of metabolic syndrome on pathologic features of prostate cancer Urol Oncol 2012 Epub ahead of print 10.1016/j.urolonc.2011.12.012 23020926

[B25] De Nunzio C Freedland SJ Miano R Trucchi A Cantiani A Carluccini A Tubaro A Metabolic syndrome is associated with high grade gleason score when prostate cancer is diagnosed on biopsy Prostate 2011 Epub ahead of print 10.1002/pros.21364 21360562

[B26] Morote J Ropero J Planas J Bastaros JM Delgado G Placer J Celma A de Torres IM Carles J Reventos J Metabolic syndrome increases the risk of aggressive prostate cancer detection BJU Int 2012 Epub ahead of print 10.1111/j.1464-410X.2012.11406.x 22883053

[B27] Post JM Beebe-Dimmer JL Morgenstern H Neslund-Dudas C Bock CH Nock N Rundle A Jankowski M Rybicki BA The Metabolic Syndrome and Biochemical Recurrence following Radical Prostatectomy Prostate Cancer 2011 2011 245642 22096652 10.1155/2011/245642 PMC3196931

[B28] Jeon KP Jeong TY Lee SY Hwang SW Shin JH Kim DS Prostate cancer in patients with metabolic syndrome is associated with low grade Gleason score when diagnosed on biopsy Korean J Urol 2012 53 9 593 597 10.4111/kju.2012.53.9.593 23060995 PMC3460000

[B29] B.K H The characteristics of prostate cancer with metabolic syndrome in Korean men Korean J Urol 2007 48 6 585 591 10.4111/kju.2007.48.6.585

[B30] Jaggers JR Sui X Hooker SP LaMonte MJ Matthews CE Hand GA Blair SN Metabolic syndrome and risk of cancer mortality in men Eur J Cancer 2009 45 10 1831 1838 10.1016/j.ejca.2009.01.031 19250819 PMC2700189

[B31] Antonio C Francesco C Cosimo DN Andrea T Rocco D Patients with metabolic syndrome and widespread high grade prostatic intraepithelial neoplasia are at a higher risk factor of prostate cancer on re-biopsy: a prospective single cohort study Urol Oncol 2012 Epub ahead of print 10.1016/j.urolonc.2012.10.004 23273912

[B32] Hammarsten J Hogstedt B Hyperinsulinaemia: a prospective risk factor for lethal clinical prostate cancer Eur J Cancer 2005 41 18 2887 2895 10.1016/j.ejca.2005.09.003 16243513

[B33] Asmar R Beebe-Dimmer JL Korgavkar K Keele GR Cooney KA Hypertension, obesity and prostate cancer biochemical recurrence after radical prostatectomy Prostate Cancer Prostatic Dis 2012 Epub ahead of print 10.1038/pcan.2012.32 PMC3860174 22907512

[B34] Lund Haheim L Wisloff TF Holme I Nafstad P Metabolic syndrome predicts prostate cancer in a cohort of middle-aged Norwegian men followed for 27 years Am J Epidemiol 2006 164 8 769 774 10.1093/aje/kwj284 16952929

[B35] Beebe-Dimmer JL Dunn RL Sarma AV Montie JE Cooney KA Features of the metabolic syndrome and prostate cancer in African-American men Cancer 2007 109 5 875 881 10.1002/cncr.22461 17265528

[B36] Higgins JP Thompson SG Quantifying heterogeneity in a meta-analysis Stat Med 2002 21 11 1539 1558 10.1002/sim.1186 12111919

[B37] Hsing AW Sakoda LC Chua S Jr Obesity, metabolic syndrome, and prostate cancer Am J Clin Nutr 2007 86 3 s843 857 18265478 10.1093/ajcn/86.3.843S

[B38] Zhang F Yang Y Skrip L Hu D Wang Y Wong C Qiu J Lei H Diabetes mellitus and risk of prostate cancer: an updated meta-analysis based on 12 case-control and 25 cohort studies Acta Diabetol 2012 Epub ahead of print 10.1007/s00592-012-0439-5 23124624

[B39] Li L Yang Y Yang G Lu C Yang M Liu H Zong H The role of JAZF1 on lipid metabolism and related genes in vitro Metabolism 2011 60 4 523 530 10.1016/j.metabol.2010.04.021 20580384

[B40] Fitzpatrick AL Daling JR Furberg CD Kronmal RA Weissfeld JL Hypertension, heart rate, use of antihypertensives, and incident prostate cancer Ann Epidemiol 2001 11 8 534 542 10.1016/S1047-2797(01)00246-0 11709272

[B41] Ganesh B Saoba SL Sarade MN Pinjari SV Risk factors for prostate cancer: An hospital-based case-control study from Mumbai, India Indian J Urol 2011 27 3 345 350 10.4103/0970-1591.85438 22022057 PMC3193734

[B42] Martin RM Vatten L Gunnell D Romundstad P Blood pressure and risk of prostate cancer: Cohort Norway (CONOR) Cancer Causes Control 2010 21 3 463 472 10.1007/s10552-009-9477-x 19949849

[B43] Discacciati A Orsini N Wolk A Body mass index and incidence of localized and advanced prostate cancer–a dose-response meta-analysis of prospective studies Ann Oncol 2012 23 7 1665 1671 10.1093/annonc/mdr603 22228452

[B44] Siegel R Naishadham D Jemal A CA Cancer J Clin 2012 62 1 10 29 10.3322/caac.20138 22237781

[B45] Jung HS Myung SK Kim BS Seo HG Metabolic syndrome in adult cancer survivors: a meta-analysis Diabetes Res Clin Pract 2012 95 2 275 282 10.1016/j.diabres.2011.08.029 22078073

